# Chemokine Receptor Activation Enhances Memory B Cell Class Switching Linked to IgE Sensitization to Alpha Gal and Cardiovascular Disease

**DOI:** 10.3389/fcvm.2021.791028

**Published:** 2022-01-13

**Authors:** Tanyaporn Pattarabanjird, Jeffrey M. Wilson, Loren D. Erickson, Lisa J. Workman, Hui Qiao, Yanal Ghosheh, Rishab Gulati, Chistopher Durant, Jenifer Vallejo, Ryosuke Saigusa, Thomas A. E. Platts-Mills, Angela M. Taylor, Klaus Ley, Coleen A. McNamara

**Affiliations:** ^1^Carter Immunology Center, University of Virginia, Charlottesville, VA, United States; ^2^Cardiovascular Research Center, University of Virginia, Charlottesville, VA, United States; ^3^Department of Biomedical Engineering, University of Virginia, Charlottesville, VA, United States; ^4^Division of Allergy and Immunology, Department of Medicine, University of Virginia, Charlottesville, VA, United States; ^5^Department of Microbiology, Immunology, and Cancer Biology, University of Virginia, Charlottesville, VA, United States; ^6^La Jolla Institute of Immunology, La Jolla, CA, United States; ^7^Division of Cardiovascular Medicine, Department of Medicine, University of Virginia, Charlottesville, VA, United States

**Keywords:** alpha-gal, B cells, coronary artery disease, IgE class switching, CITESeq

## Abstract

**Background:** Recent studies have suggested that IgE sensitization to α-gal is associated with coronary artery disease (CAD). However, the B cell subtype(s) responsible for production of IgE to α-gal and mechanisms mediating this production remain elusive.

**Methods:** Single cell multi-omics sequencing, was utilized to phenotype B cells obtained from 60 subjects that had undergone coronary angiography in whom serum IgE was evaluated by ImmunoCAP. Bioinformatics approaches were used to identify B cell subtype(s) and transcriptomic signatures associated with α-gal sensitization. *In vitro* characterization of chemokine/chemokine receptor pairs on switched memory B cells associated with IgE to α-gal was performed.

**Results:** Of the 60 patients, 17 (28%) were positive for IgE to α-gal. CITESeq identified CCR6+ class-switched memory (SWM) B cells and CXCR4 expresssion on these CCR6+ SWM B cells as significantly associated with IgE sensitization to α-gal but not to other common allergens (peanut or inhalants). *In vitro* studies of enriched human B cells revealed significantly greater IgE on SWM B cells with high CCR6 and CXCR4 expression 10 days after cells were treated with IL-4 and CD40 to stimulate class switch recombination. Both CCL20 (CCR6 ligand) and CXCL12 (ligand for CXCR4) increased the expression of IgE on SWM B cells expressing their receptors. However, they appeared to have unique pathways mediating this effect as only CCL20 increased activation-induced cytidine deaminase (AID), while CXCL12 drove proliferation of CXCR4+ SWM B cells. Lastly, correlation analysis indicated an association between CAD severity and the frequency of both CCR6+ SWM and CXCR4+ SWM B cells.

**Conclusions:** CCR6+ SWM B cells were identified as potential producers of IgE to α-gal in CAD patients. Additionally, our findings highlighted non-chemotaxis roles of CCL20/CCR6 and CXCL12/CXCR4 signaling in mediating IgE class switching and cell proliferation of SWM B cells respectively. Results may have important implications for a better understanding and better therapeutic approaches for subjects with IgE sensitization to α-gal.

## Introduction

The oligosaccharide galactose-α-1,3-galactose (α-gal) is a blood group-like oligosaccharide of non-primate mammals and the target of IgM, IgG, and IgA in all immunocompetent humans (1, 2). A subset of the population produce IgE to α-gal, and as a consequence are at risk for a syndrome of delayed anaphylaxis to mammalian meat, often called the α-gal syndrome (3). Tick bites and other parasitic exposures have been associated with α-gal IgE sensitization, however the B cell population(s) that produce this IgE and the mechanisms that lead to IgE class switch are not well-understood (4, 5). Recently our group reported that IgE sensitization to α-gal was associated with increased atheroma burden and unstable plaque characteristics in subjects undergoing evaluation for CAD (6, 7). Despite the high prevalence of α-gal sensitization in some areas and the putative connection with cardiovascular disease, the human B cell subtype that contributes to production of IgE specific for α-gal remains elusive.

To investigate human B cell subtypes that were associated with α-gal IgE sensitization, we utilized a novel single cell multi-omics sequencing platform to phenotype B cells obtained from a cohort of CAD patients with a high rate of sensitization to α-gal. CITEseq is a novel technology utilizing antibodies barcoded by DNA oligos to convert surface protein expression to sequencing readouts coupled with simultaneous mRNA sequencing (8). In conjunction with unsupervised bioinformatics pipelines, this platform allowed us to explore transcriptomic profiles and signaling signatures of human B cell subtypes associated with α-gal sensitization. Further, *in vitro* functional studies of specific B cell subtypes were performed to characterize roles of chemokine receptors in mediating IgE class switching. Identification of B cells subtypes and intrinsic signaling that contribute to α-gal sensitization will improve our understanding IgE class switch and may be helpful for future development of disease-modifying immunotherapies.

## Materials and Methods

### Human Subjects

Subjects (age range 40–80 years old) presenting to the Cardiac Catheterization laboratory at the University of Virginia (UVA) Health System, Charlottesville, Virginia, USA for a medically-indicated coronary angiogram (Coronary Assessment in Virginia cohort (CAVA) were enrolled. All participants provided written informed consent before enrollment, and the study was approved by the Human Institutional Review Board (IRB No. 15328). Peripheral blood was obtained from these participants prior to catheterization. Peripheral blood from seven volunteers was also obtained after written informed consent as part of an IRB-approved study (UVA IRB No. 16017).

### Quantitative Coronary Angiography

Patients underwent standard cardiac catheterization with two orthogonal views of the right coronary artery and four of the left coronary artery according to accepted standards. QCA was performed using automatic edge detection at an end diastolic frame. For each lesion, the frame was selected based on demonstration of the most severe stenosis with minimal foreshortening and branch overlap. Computer software was used to calculate the minimum lumen diameter, reference diameter, percent diameter stenosis, and stenosis length. Analysis was performed by blinded, experienced investigators. The Gensini score was used to assign a score of disease burden to each patient. Briefly, each artery segment is assigned a score ranging from 0 to 32 based on the percent stenosis. The severity score for each segment was multiplied by 0.5–5, depending on the location of the stenosis. Scores for all segments were then added together to given a final score of angiographic disease burden. Score adjustment for collateral was not performed for this study. Subjects with Gensini score >32 were classified as high CAD severity subjects and subjects with Gensini score ≤ 6 were classified as low CAD severity subjects. Clinical characteristics of the CAVA cohort subjects used in this study were provided in Supplementary Table 1.

### Quantification of Serum IgEs in Humans

Serum IgE to α-Gal, dust mite (*Dermatophagoides pteronyssinus*), oak, timothy grass, and peanut were assayed using ImmunoCAP 250 (Thermo-Fisher/Phadia, Kalamazoo, MI). Of note, the α-gal IgE assay used here was the commercial ImmunoCAP which uses beef thyroglobulin on the solid phase. Subjects with IgE to α-Gal >0.1 kU_A_/L were classified as α-Gal sensitized subjects. Subjects with IgE to inhalants and peanuts >0.35 kU_A_/L were classified as inhalants or peanuts sensitized subjects.

### PBMC Isolation

Blood from enrollees was drawn into BD K2 EDTA vacutainer tubes and processed at room temperature (RT) within one hour of collection. Whole blood in vacutainers were centrifuged at 400 x g for 10 min at RT to remove platelet rich plasma. Plasma was cryopreserved at −80°C freezer. PBMCs were isolated by Ficoll-Paque PLUS (GE Healthcare Biosciences AB) gradient centrifugation using SepMate-50 tubes (Stemcell Technologies Inc.) following the manufacturer's protocol. Trypan blue staining of PBMCs was performed to quantify live cell counts. PBMCs were cryopreserved in freezing solution (90% FBS/10% DMSO), or used fresh in assays. PBMC vials were stored in Mr. Frosty (Thermo Fisher) for 48 h at −80°C and were then stored in liquid nitrogen until used.

### CITESeq Optimization and Staining

PBMCs obtained from 60 CAVA subjects were labeled with the BD Single-Cell Multiplexing Kit (BD Biosciences) and CITESeq antibody-oligomers (Ab-Oligos) reagents following the protocol outlined by Vallejo et al. (9). Briefly, PBMC vials were thawed at 37°C and then washed with complete RPMI-1640 solution. Cells were aliquoted at 1 million cells per tube and incubated on ice with Fc Block (BD Biosciences) at a 1:20 dilution in super bright staining buffer (SB, eBioscience) and subsequently transferred to multiplexing kit tubes (BD Biosciences) and incubated for 20 min at RT. Cells were then washed 3 times and centrifuged at 400 x g for 5 min. DRAQ7 and Calcein AM were used to quantify cell viability. Tube contents were pooled in equal proportions with total cell counts not to exceed 1 million cells, and resuspended in 20 μL of SB with 50 unique CITESeq Ab-Oligos diluted at 2 μL each as listed in Supplementary Table 2. The pooled samples were then incubated on ice for 30–60 min, washed with 2 mL of SB, and centrifuged at 400 x g for 5 min per manufacturer's recommendations. This step was repeated two more times for a total of three washes. The cells were then counted again using BD Rhapsody Scanner.

### Library Preparation

Cells were loaded at 800–1,000 cells/μL into the primed plate. Reverse Transcription was performed at 37°C on a thermomixer at 1,200 rpm for 20 min following with addition of Exonuclease I with 30 min incubation on a thermomixer and then transferred to heat block to incubate at 80^o^C for 20 min. BD's protocol was used to prepare the cDNA library as described in Vallejo et al. (9). The final QC and quantification check were performed using TapeStation and Qubit kits and reagents.

### Sequencing

The samples were pooled and sequenced to the following depth recommended by BD: Ab-Oligos sequencing: 40,000 reads per cell; mRNA: 20,000 reads per cell; Sample Tags: 600 reads per cell. A total of 60,600 reads per cell were obtained for sequencing on the NovaSeq. The samples and specifications for pooling and sequencing depth, along with number of cells loaded onto each plate was optimized for S1 and S2 100 cycle kits (Illumina). Once sequencing was complete, the FASTA file and FASTQ files generated by the NovaSeq were uploaded to Seven Bridged Genomics pipeline, where the data was filtered in matrices and csv files. Doublet Finder package on R (https://github.com/chris-mcginnis-ucsf/DoubletFinder) was used to remove the doublets and cells with <128 antibody molecules sequenced were removed. All antibody sequencing data were CLR (centered log-ratio) normalized and converted to log2 scale. All transcripts were normalized by total UMIs in each cell and scaled up to 1,000.

### CITEseq Data Pre-processing and Analysis

B cells within PBMCs obtained from CAVA subjects were first identified by manual gating determined by CD19^+^CD3^−^ antibody sequencing and thresholding of each antibody expression was determined based on expression on negative cells. UMAP dimensionality reduction and Louvain clustering on 26 antibodies B cell markers using the python scanpy package (https://github.com/theislab/scanpy/issues/350) were performed to subtype B cells. Expression of the 488 genes within B cell subtypes were compared between subjects with and without α-gal sensitization by performing *t*-test with Bonferonni corrected *p*-values to obtain false discovery rate (FDR) values and calculating fold change in gene expression by using R software. Differentially expressed genes were defined as genes with FDR < 0.05 and Log_2_FC < 1 or > 1. Volcano plots of differentially expressed genes were visualized by using the python bioinfokit package. Ingenuity Pathway Analysis was performed on all the annotated RNA to analyze for differentially regulated cellular processes and canonical pathways.

### Flow Cytometry Characterization of B Cells Obtained From CAVA Subjects

Cryopreserved PBMCs from 10 CAVA subjects (with and without α-gal sensitization) were thawed, washed twice with warm complete media (RPMI with L-Glutamine supplemented with 5% heat-inactivated human AB serum (Sigma), 1 mM sodium pyruvate, 0.01 M HEPES, 1x MEM non-essential amino acids, 50 μM 2-Mercaptoethanol, and 1 mM Pen-Strep (all from Gibco). Total B cells were enriched using the STEMCELL human B cell enrichment kit (StemCell Technologies). IgE-expressing B cells were identified and characterized using flow cytometry with mAbs as listed in Supplementary Table 3. Cells were acquired on Cytek Aurora and the analysis was performed using FCS Express 7. The gating strategy is shown in Supplementary Figure 1.

### *In vitro* Stimulation of B Cells

Cryopreserved PBMCs from volunteers were thawed, washed twice and enriched for total B cells as described in section Flow Cytometry Characterization of B cells obtained from CAVA subjects. Enriched B cells were cultured in 200 μL of complete RPMI with 20 ng/mL human IL-4 (R&D system), ± 10 μg/mL agonistic anti-human CD40 mAb (ThermoFisher; clone 5C3), ± 20 ng/mL human CCL20 (R&D system), ± 100 ng/mL human CXCL12 (R&D system) ± 10 nM CCR6 inhibitor 1 (MedChemExpress) ± 5 uM AMD3100 (Sigma) for 10 days in Corning Costar 96-well flat bottom plates. Levels of proliferation and immunoglobulin class switching to IgG and IgE produced by human B cells were determined, before and up to 10 days after stimulation, by using flow cytometry with mAbs as listed in Supplementary Table 3. Cells were acquired on Cytek Aurora and the analysis was performed using FCS Express 7. The gating strategy is presented in Supplementary Figure 1.

### Statistics

Statistics were calculated using GraphPad Prism Version 7.0a (GraphPad Software, Inc.), Python 3.0, R 3.6.1 or SAS 9.4. Results from all replicated experiments are displayed, and bar graphs display mean ± SEM.

## Results

### Single Cell Multi-Omics Platform Identified an Association Between Circulating CCR6+ Switched Memory B Cells and **α**-gal IgE Sensitization

**Figure 1:**
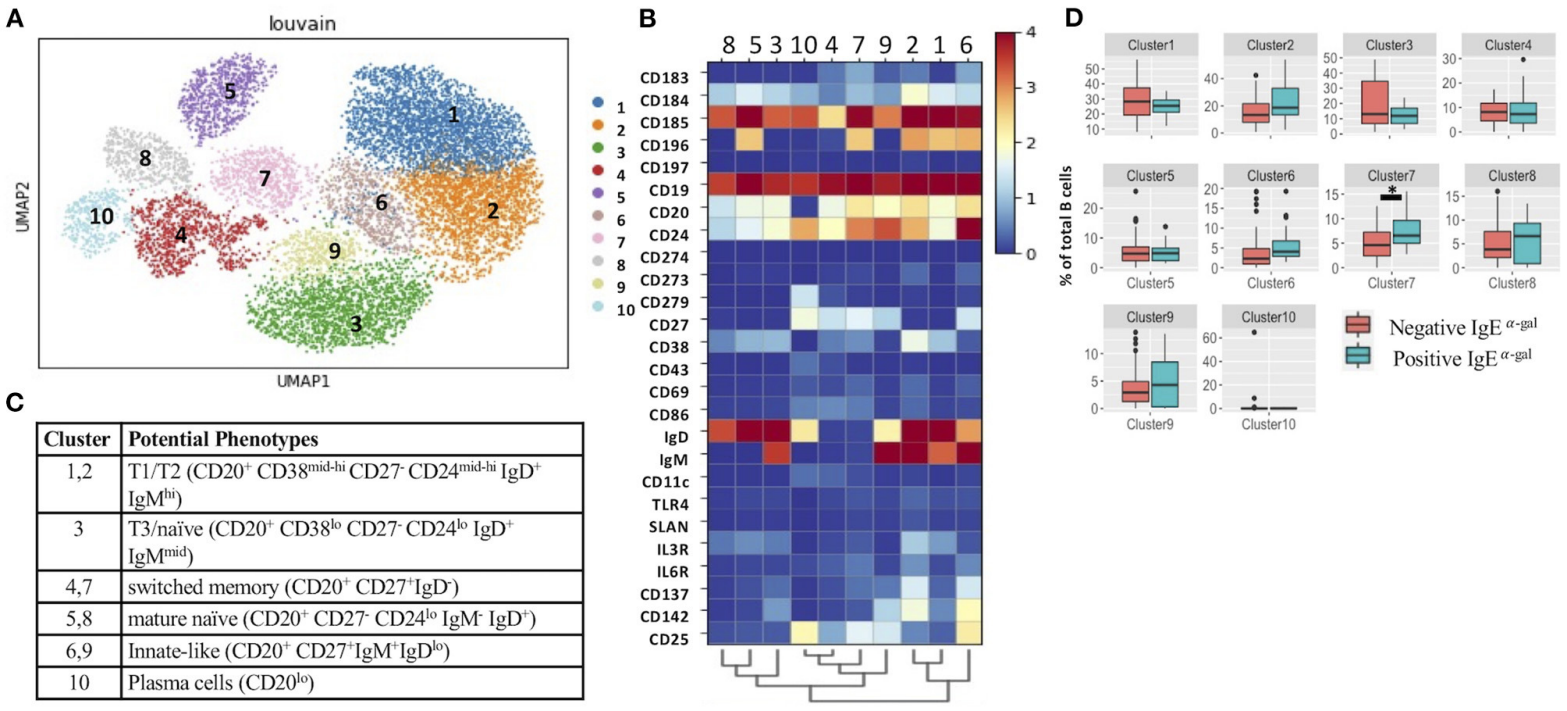
Single cell multi-omics platform identified an association between circulating CCR6+ switched memory B cells and α-gal sensitization. **(A)** Representative metacluster UMAP showing 10 distinguished B cell subsets by using Louvain clustering. **(B,C)** Heatmap showing median expression of surface markers from metaclustering **(B)**, table showing potential phenotypes of each B cell subset resulting from Louvain clustering **(C)**. **(D)** Biaxial plots to compare frequency of each cluster as a percentage of total B cells in subjects with and without α-gal sensitization. *p < 0.05 by Mann-Whitney test.

Consistent with a prior investigation that showed a high prevalence of α-gal IgE sensitization in the CAVA cohort (6), α-gal IgE was detected in 17 of the 60 patients (28%). Demographics of these patients are provided in Supplementary Table 1. To explore associations between α-gal sensitization and B cell subsets, we applied CITEseq with a 50-antibody panel on B cells obtained from 60 subjects in CAVA cohort. Among CD19+ B cells, 26-B cell antibodies were used in combination with metalouvain clustering to reveal 10 distinct B cell clusters within the PBMCs (Figure 1A). Potential phenotypes for each cluster were assigned based on expression of distinguishing B cell markers as represented in the heatmap (Figures 1B,C). Of all the clusters, only cluster 7 had elevated frequency in subjects with α-gal sensitization (Figure 1D). This cluster had features of a switched memory (SWM) B cell based on the expression profile of CD20+/CD27+/IgD-. Interestingly, IgE to other representative food (peanut) and aero-allergens (dust mite, oak, and timothy grass), did not have a significant relationship with frequency of any circulating B cell clusters (Supplementary Figure 2). Cluster 4 and 7 shared several markers, but CCR6 was highly expressed in cluster 7 but not in cluster 4 (Figures 1B,C). This result suggested that α-gal sensitization was specifically associated with SWM B cells that expressed CCR6. To further explore relationship between CCR6+ SWM B cells and IgE α-gal sensitization, we obtained B cells from CAVA subjects to compare percentages of IgE expressing SWM B cells in subjects with and without α-gal IgE sensitization. We found higher percentage of IgE+ SWM in subjects positive for α-gal IgE compared to those that were negative (Supplementary Figure 3A). In addition, higher percentage of IgE expressing CCR6+ SWM but not CCR6- SWM was observed in α-gal sensitized subjects (Supplementary Figure 3B).

### CXCR4 Is Enriched in CCR6+ Switched Memory B Cells of **α**-gal Sensitized Subjects

**Figure 2:**
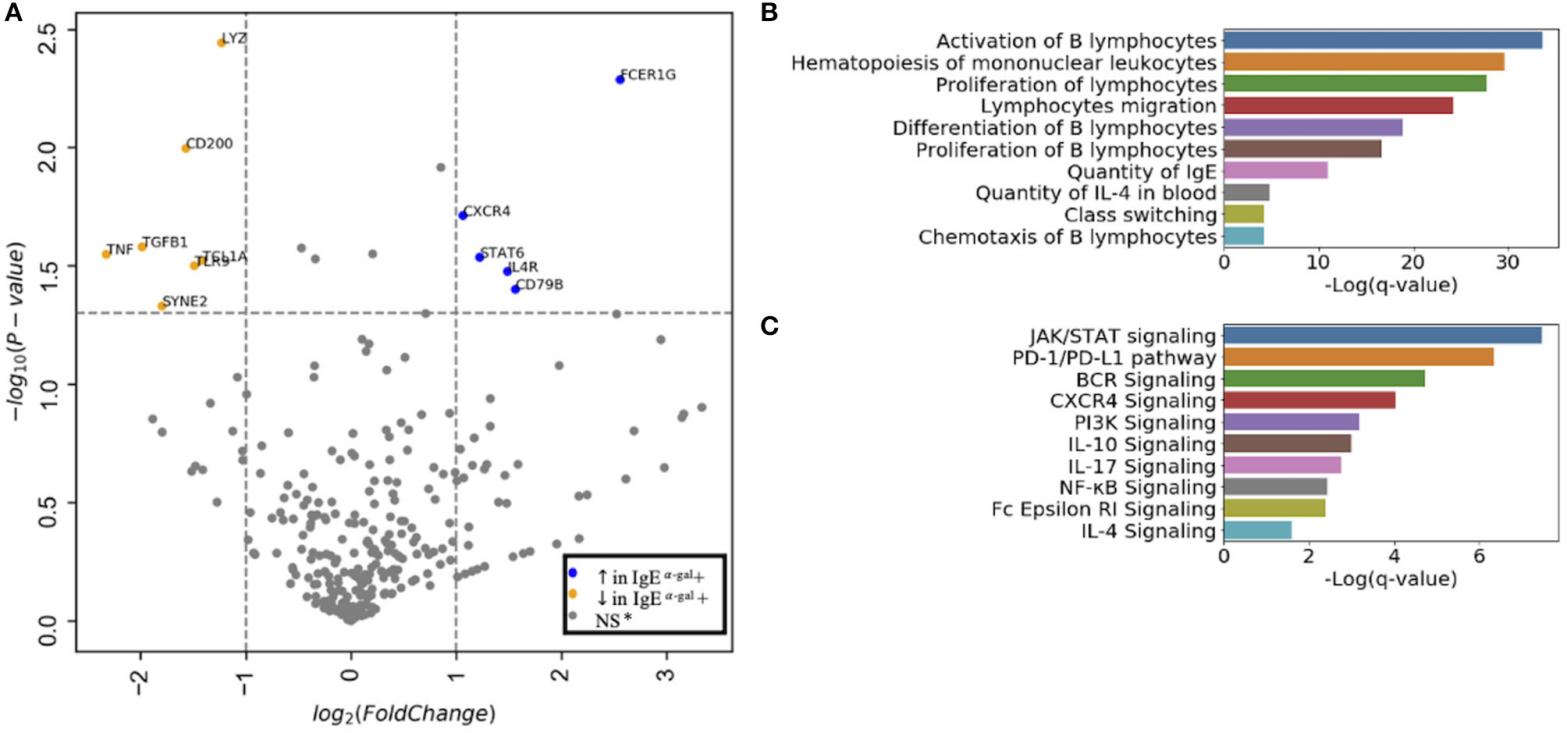
CXCR4 signaling is enriched in CCR6+ SWM B cells of α-gal sensitized subjects. **(A)** Volcano plot of 488 gene array demonstrating DEGs of cluster 7 upregulated (blue) and downregulated (orange) in IgE α-gal + subjects. (**B,C)** Significant cellular processes **(B)** and canonical pathways **(C)** enriched in IgE α-gal + in cluster 7 cells. ns, non-significant.

Next, we evaluated cluster 7 using a 488 immune-relevant CITEseq transcriptomic sequencing to identify differentially expressed genes (DEGs) between α-gal sensitized and non-sensitized patients. Using an FDR of <0.05, CXCR4, STAT6, IL4R, CD79B and FCER1G were enriched in cluster 7 of subjects with detectable levels of IgE to α-gal (Figure 2A). Ingenuity pathway analysis of DEGs with an FDR cutoff of 0.1 indicated cellular processes such as B cell activation, proliferation, class switching, chemotaxis and IgE production enriched in subjects with α-gal IgE sensitization (Figure 2B). Canonical pathways dominated by activating pathways such as JAK/STAT, BCR, CXCR4, PI3K, NFKB, and IL-4 signaling (Figure 2C) were enriched in subjects with α-gal sensitization. Of note, a known inhibitory pathway, PD1/PDL1 was also enriched in cluster 7 of subjects with α-gal sensitization.

**Figure 3:**
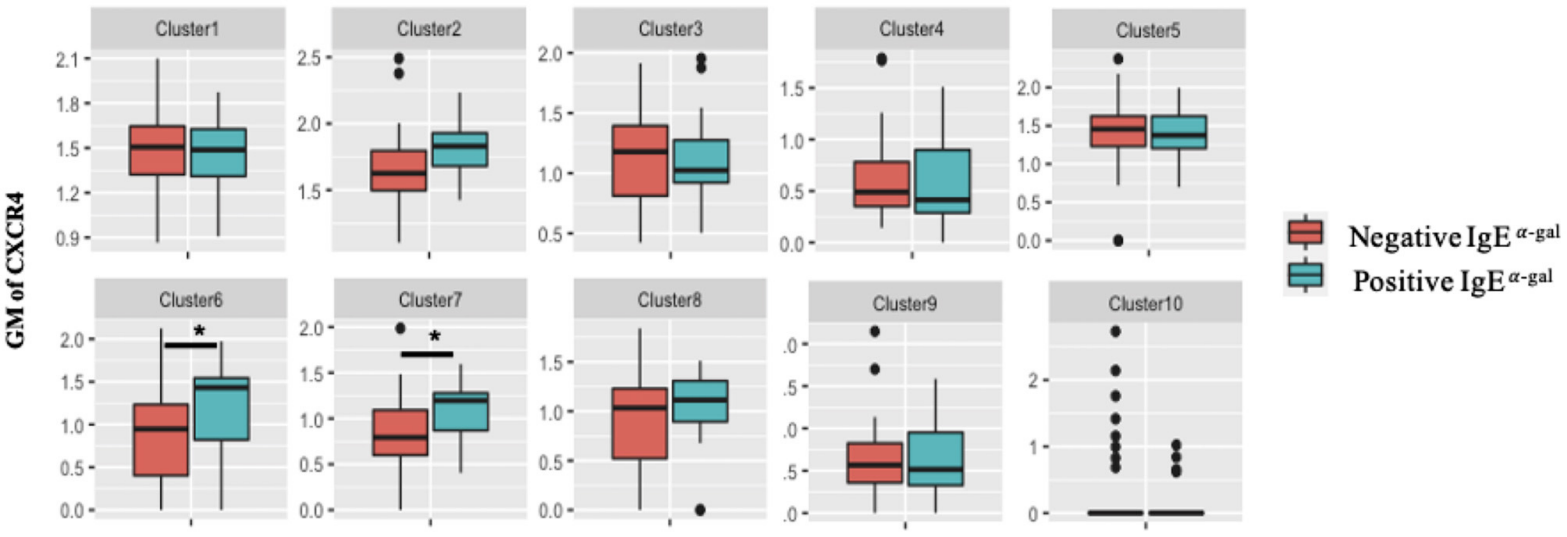
CXCR4 surface expression increases in CCR6+ SWM B cells of α-gal sensitized subjects. Biaxial plots to compare geometric means (GM) of CXCR4 across 10 B cell clusters between subjects with and without α-gal sensitization. *p < 0.05 by Mann-Whitney test.

A role for BCR activation, class switch recombination machinery and IL-4 signaling are well-documented in IgE class switching (10–12). Here, we further explored roles of chemokine receptors in contributing to B cell IgE class switching. As CXCR4 transcript expression in cluster 7 was enriched in α-gal sensitized subjects (Figure 2A), surface protein expression of CXCR4 on all 10 B cell clusters was investigated. Using the 26-surface antibody CITEseq panel, CXCR4 levels were evaluated using geometric mean (GM) and compared between α-gal sensitized and non-sensitized subjects. Of all the 10 clusters, enrichment of CXCR4 surface expression in α-gal sensitized subjects was only observed in cluster 6 and 7 (Figure 3). No difference in CXCR4 surface expression was observed in relationship to peanut or inhalant allergens sensitization (Supplementary Figure 4).

### CCR6 and CXCR4 Are Important for IgE Isotype Switching

**Figure 4:**
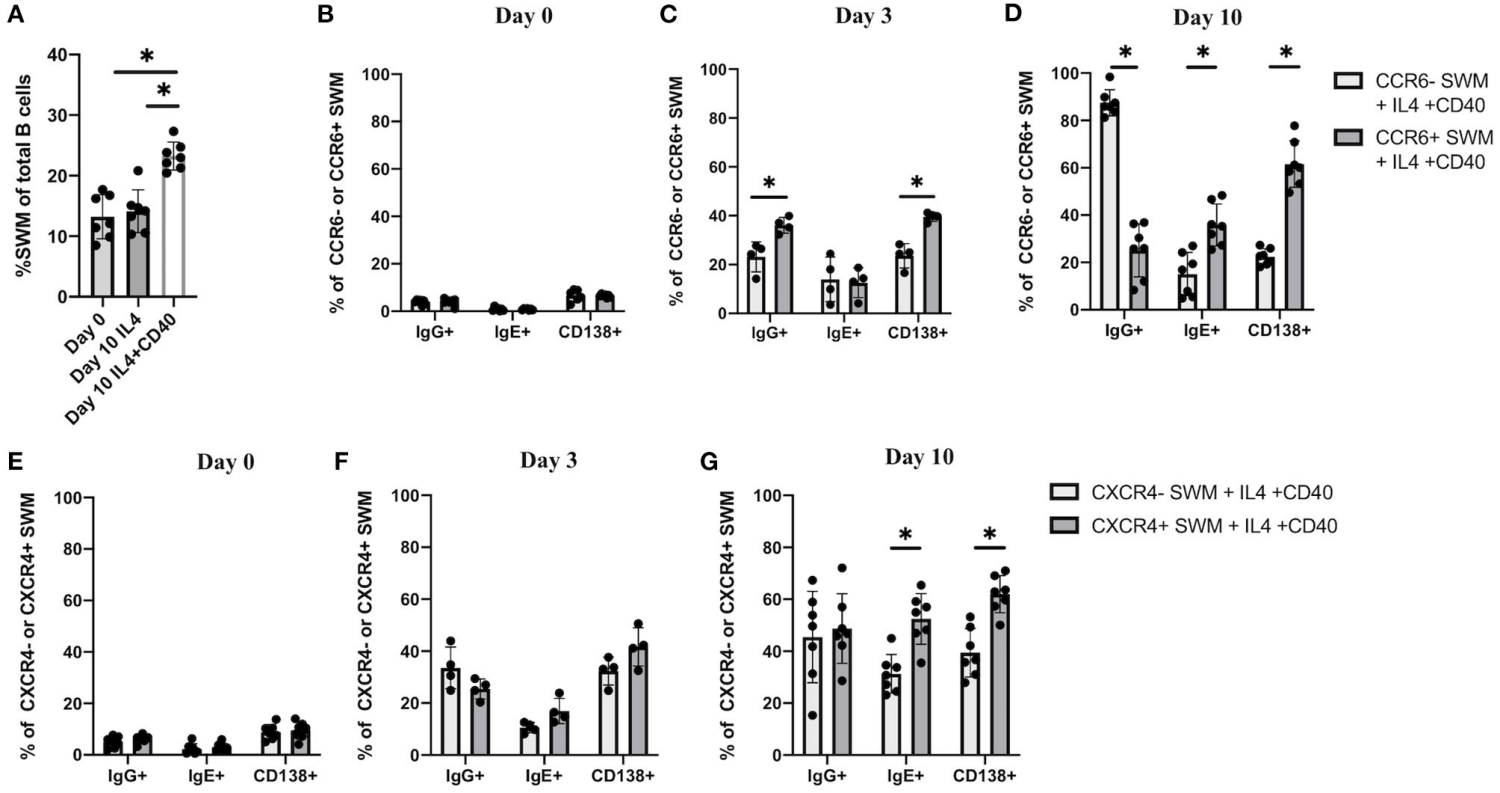
CCR6 and CXCR4 are important for immunoglobulin isotype switching. **(A)** Percentage of SWM of total B cells at day 0 before stimulation and at day 10 post with 20 ng/mL IL-4 and ± 10 ug/mL anti-CD40 stimulation. **(B–D)** Percentage of IgG+, IgE+, and CD138+ of CCR6- SWM or CCR6+ SWM at day 0 **(B)**, day 3 **(C)**, and day 10 **(D)** after culturing with 20 ng/mL IL-4 and 10 ug/mL anti-CD40. **(E–G)** Percentage of IgG+, IgE+, and CD138+ of CXCR4- SWM or CXCR4+ SWM at day 0 **(E)**, day 3 **(F)**, and day 10 **(G)** after with 20 ng/mL IL-4 and 10 ug/mL anti-CD40. *p < 0.05 by Mann-Whitney test.

The combination of IL-4 and CD40 signaling are known to induce immunoglobulin class switching (13, 14). Here we performed *in vitro* culture of human B cells obtained from volunteers to further investigate IgE class switching in relation to CCR6 and CXCR4 expression. B cells treated for 10 days with IL-4/anti-CD40 had a higher percentage of SWM B cells compared to baseline and as compared to day 10 cells treated with IL-4 stimulation alone (Figure 4A). We subsequently evaluated expression of IgG, IgE, and the plasma cell marker CD138, with flow cytometry at day 0, 3 and 10 of culture (Figures 4B–D). Percentages of SWM B cells expressing IgG, IgE, and CD138 were low regardless of CCR6 expression at day 0 (Figure 4B) and increased at day 3 and 10 (Figures 4C,D). IgG and CD138, but not IgE, were expressed at higher levels on CCR6+ compared to CCR6- SWM B cells at day 3 (Figure 4C). However, at day 10 percentages of CCR6+ SWM B cells expressing IgE were higher when compared to the earlier time point and to CCR6- SWM B cells (Figure 4D). Interestingly, percentages of CCR6+ SWM B cells expressing IgG were lower compared to CCR6- SWM B cells at day 10 after stimulation (Figure 4D). Next, we continued our analysis of CXCR4- and CXCR4+ SWM B cells. We found low percentages of SWM B cells expressing surface IgG, IgE, and CD138 regardless of CXCR4 expression before stimulation (Figure 4E), which increased after stimulation for 3 days (Figure 4F). However, no significant differences among these populations were observed between CXCR4- and CXCR4+ SWM B cells. After 10 days, the CXCR4+ SWM B cells showed a significant increase in surface IgE and CD138 as compared to earlier timepoints and as compared to CXCR4- SWM B cells (Figure 4G). The results altogether suggested that greater class switching to IgE was observed in CCR6+ and CXCR4+ SWM B cells.

### CCL20/CCR6 Intrinsic Signaling Augments IgE Isotype Switching Through Induction of AID Expression

**Figure 5:**
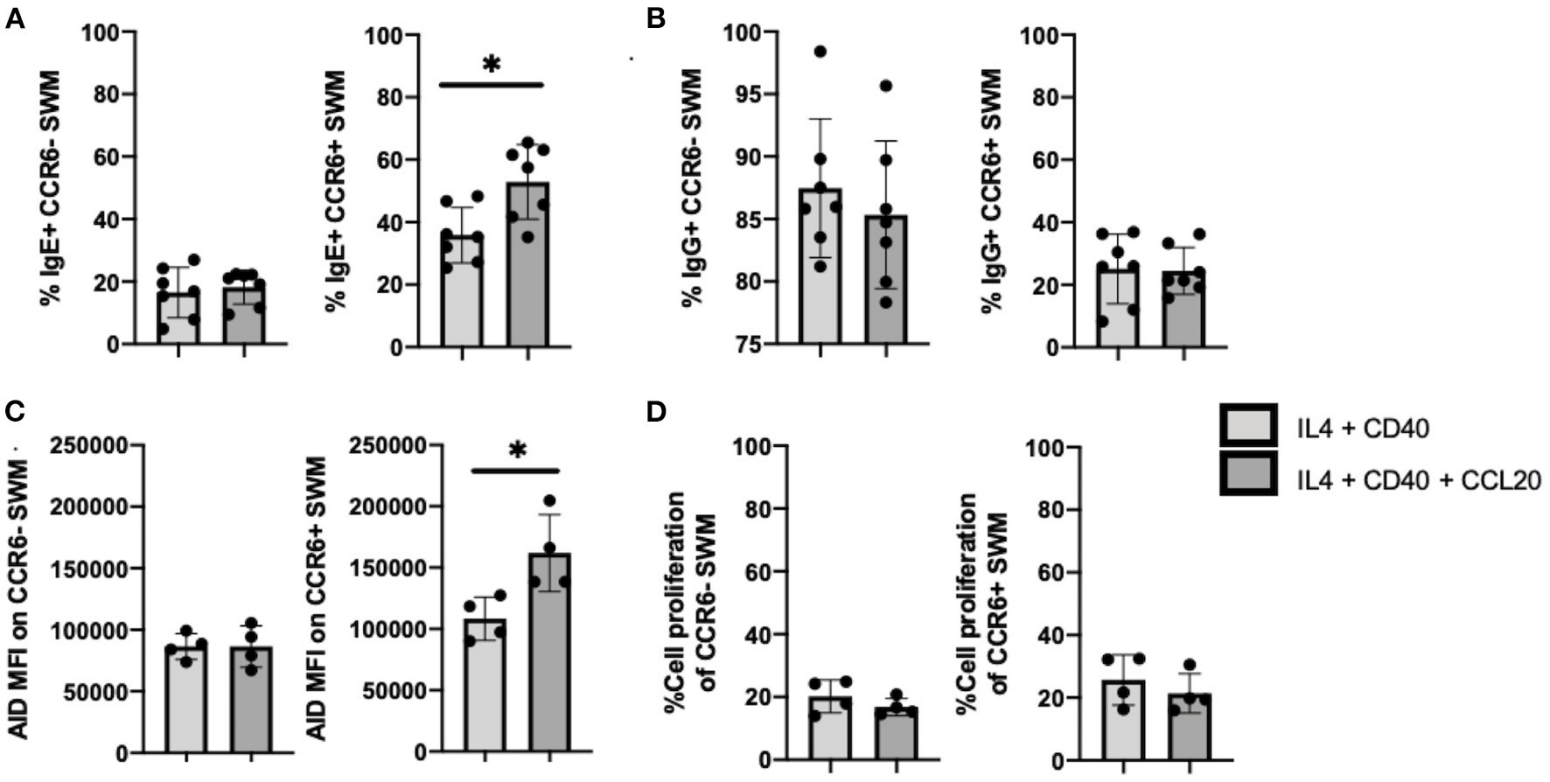
CCL20/CCR6 intrinsic signaling augments IgE isotype switching through induction of AID expression. **(A)** Percentage of IgE+ of CCR6- SWM and CCR6+ SWM after 10 days culture with 20 ng/mL IL-4, 10 ug/mL anti-CD40 ± 20 ng/mL CCL20. **(B)** Percentage of IgG+ of CCR6- SWM and CCR6+ SWM after 10 days culture with 20 ng/mL IL-4, 10 ug/mL anti-CD40 ± 20 ng/mL CCL20. **(C)** Expression of AID measured by MFI on CCR6- SWM and CCR6+ SWM after 3 days culture with 20 ng/mL IL-4, 10 ug/mL anti-CD40 ± 20 ng/mL CCL20. **(D)** Percentage of cell proliferation measured by cell-trace violet of CCR6- SWM and CCR6+ SWM after 3 days culture with 20 ng/mL IL-4, 10 ug/mL anti-CD40 ± 20 ng/mL CCL20. *p < 0.05 by Mann-Whitney test.

Further, we investigated the roles of CCL20/CCR6 intrinsic signaling in mediating immunoglobulin isotype switching by using CCL20 along with IL-4 and anti-CD40 to stimulate enriched human B cells from volunteers. After 10 days of culture, IgE expression was enhanced in CCL20 treated CCR6+, but not CCR6- SWM B cells (Figure 5A). No differences in IgG surface expression were observed with CCL20 stimulation in either of CCR6- or CCR6+ SWM (Figure 5B). These results suggested that intrinsic CCL20/CCR6 signaling in SWM B cells contributed to IgE class switching. As AID enzyme is known to induce immunoglobulin isotype switching (15), expression of AID was measured both in CCR6- and CCR6+ SWM under conditions of IL4 and anti-CD40 treatment ± CCL20. After 3 days of culture, CCL20 augmented AID expression in the CCR6+ SWM but not the CCR6- SWM B cells (Figure 5C; Supplementary Figure 5), while no change in cell proliferation was observed (Figure 5D). These findings suggested that CCL20/CCR6 intrinsic signaling induced AID expression.

### CXCL12/CXCR4 Signaling Drives Proliferation of SWM B Cells

**Figure 6:**
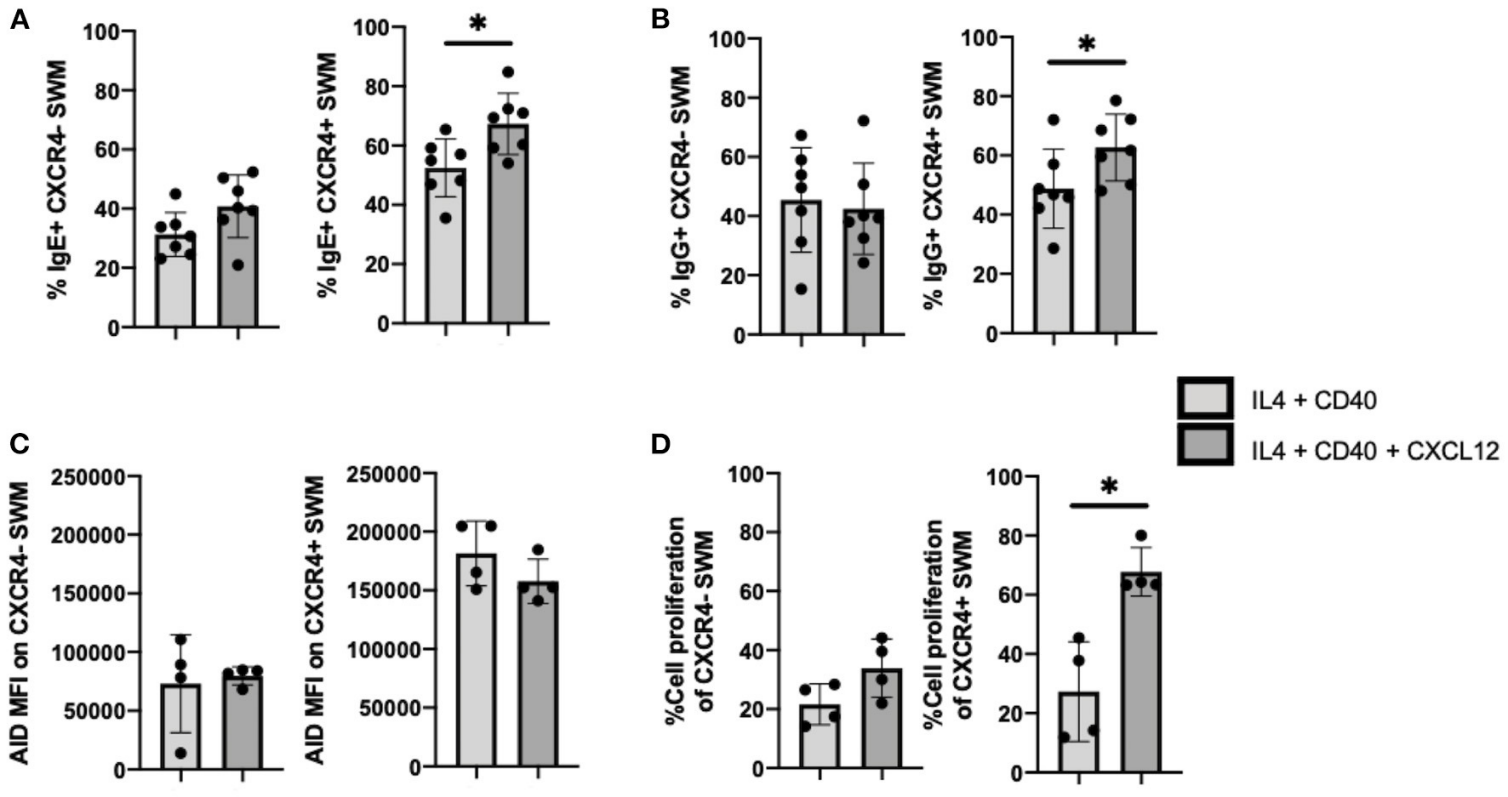
CXCL12/CXCR4 intrinsic signaling drives proliferation of SWM B cells. **(A)** Percentage of IgE+ of CXCR4- SWM and CXCR4+ SWM after 10 days culture with 20 ng/mL IL-4, 10 ug/mL anti-CD40 ± 100 ng/mL CXCL12. **(B)** Percentage of IgG+ of CXCR4- SWM and CXCR4+ SWM after 10 days culture with 20 ng/mL IL-4, 10 ug/mL anti-CD40 ± 100 ng/mL CXCL12. **(C)** Expression of AID measured by MFI on CXCR4- SWM and CXCR4+ SWM after 3 days culture with 20 ng/mL IL-4, 10 ug/mL anti-CD40 ± 100 ng/mL CXCL12. **(D)** Percentage of cell proliferation measured by cell-trace violet of CXCR4- SWM and CXCR4+ SWM after 3 days culture with 20 ng/mL IL-4, 10 ug/mL anti-CD40 ± 100 ng/mL CXCL12. *p < 0.05 by Mann-Whitney test.

To follow up on our observation that IgE expression occurred preferentially in CXCR4+ SWM B cells (Figure 4G), we further investigated intrinsic roles of CXCL12/CXCR4 in mediating immunoglobulin class switching. CXCL12 stimulation did not affect IgE surface expression of CXCR4- SWM, however CXCR4+ SWM cells had a greater frequency of IgE expression after 10 days culture (Figure 6A). Similarly, CXCL12 did not alter IgG surface expression of CXCR4- SWM yet did enhance the percentages of IgG expression in CXCR4+ SWM B cells (Figure 6B). Effects of CXCL12 on AID expression and cell proliferation of CXCR4- and CXCR4+ SWM B cells were also evaluated. The result indicated no change in AID expression (Figure 6C; Supplementary Figure 6), but we did observe an increase in cell proliferation in CXCR4+ SWM cells after CXCL12 stimulation (Figure 6D). These results suggest that CXCL12/CXCR4 intrinsic signaling drove proliferation of SWM B cells, not class switching, leading to higher percentages CXCR4+ SWM with IgG and IgE expression.

### Chemokine Receptor Inhibitors Reduce IgE Isotype Switching

**Figure 7:**
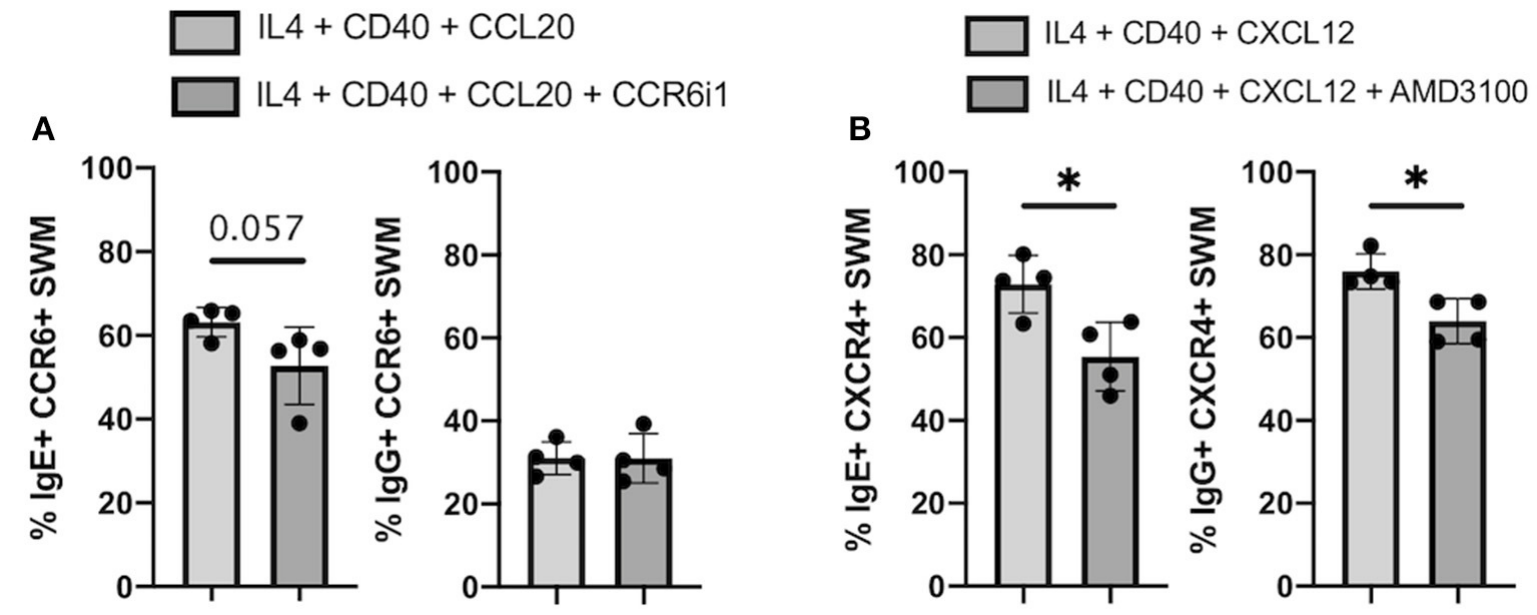
Chemokine receptor inhibitors reduce IgE isotype switching. **(A)** Percentage of IgE+ and IgG+ of CCR6+ SWM after 10 days culture with 20 ng/mL IL-4, 10 ug/mL anti-CD40 ± 20 ng/mL CCL20 ± 5uM CCR6i1. **(B)** Percentage of IgE+ and IgG+ of CXCR4+ SWM after 10 days culture with 20 ng/mL IL-4, 10 ug/mL anti-CD40 ± 100 ng/mL CXCL12 ± 5 uM AMD3100. *p < 0.05 by Mann-Whitney test.

CCR6 inhibitor 1 (CCR6i1) (16) and AMD3100 (17) were previously shown to be selective inhibitors for blocking CCL20/CCR6 and CXCL12/CXCR4 signaling, respectively. Here, we utilized these inhibitors to evaluate the impacts of blocking these chemokine receptor pathways on immunoglobulin isotype switching. After 10 days of culture, we found that the percentage of CCL20-stimulated CCR6+ SWM B cells expressing IgE trended to be lower with CCR6i1 treatment, whereas CCR6i1 had no impact on the percentage of IgG SWM B cells (Figure 7A). AMD3100 treatment of CXCR4+ SWM B cells stimulated with CXCL12 yielded fewer IgE and IgG expressing SWM cells (Figure 7B).

### CXCR4 and CCR6 Expression on SWM B Cells Directly Associates With CAD Severity

**Figure 8:**
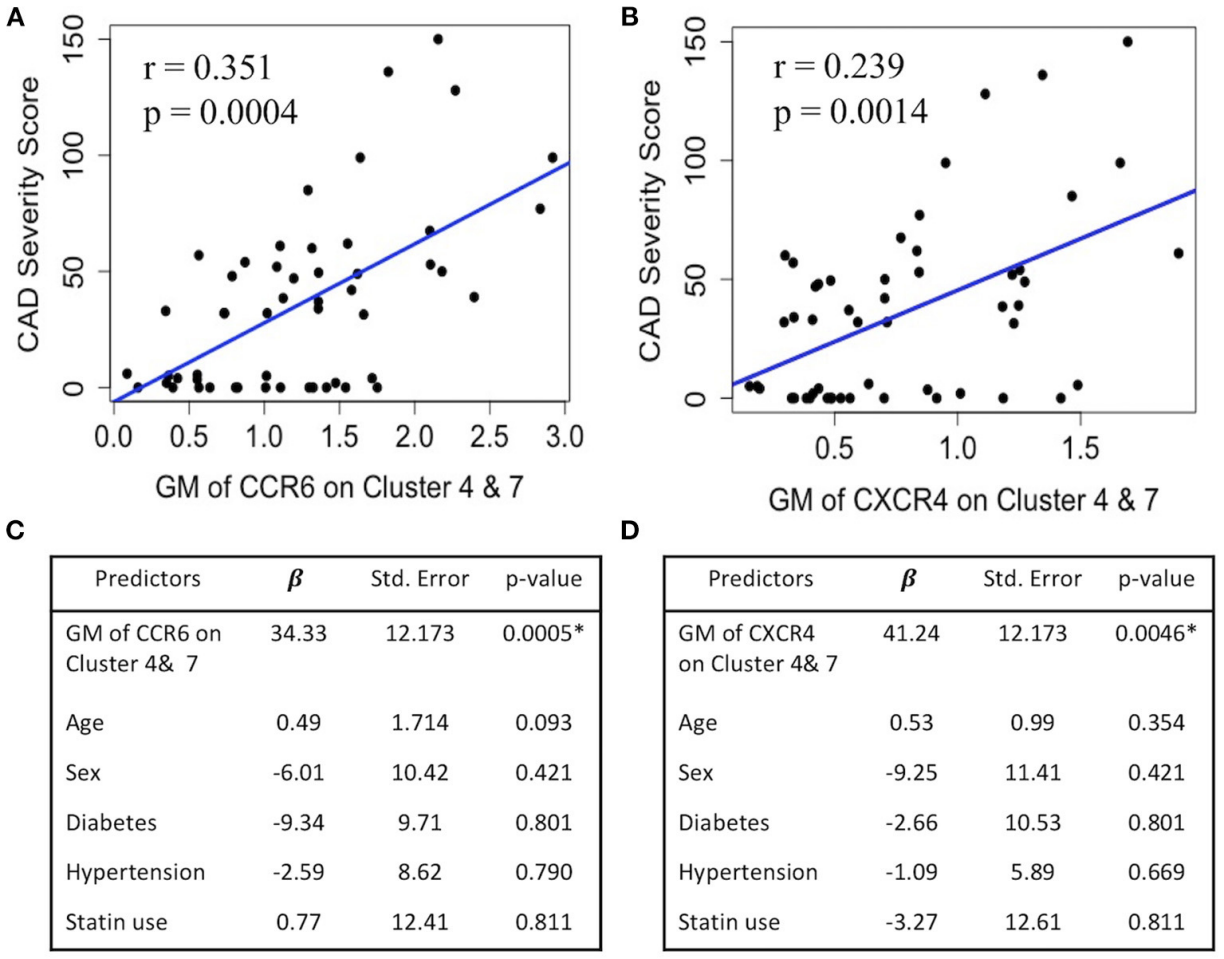
CXCR4 and CCR6 expression on SWM B cells directly associates with CAD severity. **(A,B)** Linear regression between GM of CCR6 **(A)** and CXCR4 **(B)** on cluster 4 and 7 and CAD Gensini severity score. **(C,D)** Multivariate regression between GM of CCR6 **(C)** and CXCR4 **(D)** on cluster 4 and 7 and CAD Gensini severity score with age, sex, diabetes status, hypertension and statin use as covariates. *p < 0.05 by Mann-Whitney test.

Because CCR6 and CXCR4 on SWM B cells potentially play a role in mediating α-gal sensitization (Figures 1D, 2A) and α-gal sensitization has previously been associated with CAD (6), here we explored the relationship between CCR6/CXCR4 expression on SWM B cells and atherosclerosis. In this study CAD severity was evaluated by quantitative coronary angiography (QCA) and quantified by the well-established Gensini scoring system (18). While the frequency of SWM B cells did not associate with CAD (data not shown), the expression of both CCR6 (*R*^2^ = 0.35, *P* < 0.001) and CXCR4 (*R*^2^ = 0.24, *p* = 0.001) on SWM B cells demonstrated significant correlations with CAD severity in linear regression analysis (Figures 8A,B). The correlations between CCR6 and CXCR4 on SWM B cells and CAD severity remained significant when using cardiac risk factors as co-variates (Figures 8C,D).

## Discussion

Through utilization of CITEseq, here we identified a B cell cluster uniquely marked by CCR6+ CD20+/CD27+/IgD-/IgM- to associate with α-gal sensitization in CAVA subjects. CD27+/IgD- B cells can be traditionally classified as SWM B cells (19). CD27+ memory B cells harness somatically mutated BCR (20–22) and represent heterogeneous populations that differ in expression of the immunoglobulin isotypes IgM, IgG, IgA and IgE. Human IgE+ B cells have been long thought to mainly develop indirectly from IgG1+ B cell intermediates and rarely directly from IgM+ or IgD+ B cells (23, 24). This premise has been supported by recent studies which used deep sequencing of immunoglobulin somatic hypermutations (25). Roles of chemokine receptors in contributing to IgE production have also been studied in murine models. CCR6 knockout mice were previously shown to have lower serum IgE in hypersensitivity models (26). A study of patients with red meat allergy by Cox et al. reported that a CCR6+ B cell population produced IgE to α-gal (27). Additionally, inflamed skin at the site of tick antigen exposure also demonstrated the accumulation of high levels of CCR6^high^ B cells (28). Our study is the first to show that individuals with IgE sensitization to α-gal without diagnosed red meat allergy (α-gal syndrome) have high expression of CCR6 on SWM B cells. Taken together, our current findings and prior literature support the premise that CCR6+ class-switched memory B cells are a source of B cells that produce allergen-specific IgE, including IgE to α-gal. Moreover, we demonstrate that CCR6 and CXCR4 expression on SWM B cells is higher on those with severe CAD.

Within CCR6+ SWM, transcriptome analysis also revealed that high levels of CXCR4, STAT6, IL-4R, and CD79B (BCR co-receptor) expression associated with α-gal sensitization. IL-4 signaling in B cells has been recognized as a significant driver for IgE class switching through phosphorylation of STAT6 (11, 12). In addition, in agreement with our data indicating an increase in CD79B expression in alpha-gal sensitized subjects, IgE expression on B cells has been previously shown to be dependent on CD79 (10). To our knowledge, a role for CXCR4 in promoting B cell production of IgE has not been reported, but here we found that CXCR4 transcripts and protein expression were associated with α-gal sensitization.

More importantly, this study suggests that CCR6 and CXCR4 contribute to IgE class switch via mechanisms unrelated to chemotaxis. Traditionally, CCR6 and CXCR4 are known to promote migration and entry of B cells to germinal center (GC) regions where B cells class-switch and differentiate (29, 30). Here, we demonstrate for the first time that intrinsic signaling of CCL20/CCR6 can augment AID expression and facilitate IgE isotype switching. This IgE class switching potentially occurs indirectly via an IgG+ B cell given the frequency and kinetics observed over the 10-day experiment. However, future studies will be required to further characterize the mechanism whereby IgE is augmented.

Additionally, we also describe a novel role for CXCL12/CXCR4 signaling in contributing to the proliferation of SWM B cells. Non-chemotaxis roles for CXCR4 are not unprecedented. CXCR4 has been previously shown to promote the survival of a human B lymphoblast cell line in the setting of oxidative stress (31). The current study emphasizes that CXCR4 promotes proliferation of B cells in addition to regulating B cell survival as previously shown in the literatures (31, 32). However, signaling pathways that mediate CXCL12/CXCR4-induced proliferation are still unexplored and investigation to show whether CXCR4 can promote proliferation of other B cell subtypes apart from SWM B cells is still required.

As α-gal IgE sensitization has been shown to associate with CAD (6, 7), we further explored roles of CCR6 and CXCR4 in cardiovascular disease. Upadhye et al. and Srikakulapu et al. had previously investigated roles of CCR6 and CXCR4 on B cells in contributing to atherosclerosis (33, 34). However, those studies focused on atheroprotective B-1 cells and the role of CCR6 and CXCR4 in boosting anti-inflammatory IgM production. This study for the first time suggests a role for CCR6 and CXCR4 on SWM B cells that could be atherogenic. The SWM B cell is a subtype of B-2 cells which have been shown in murine studies to be atherogenic mainly through differentiation into plasma cells and production of pro-inflammatory IgG antibodies (35). In humans, nevertheless, the relationship between SWM B cells, IgG production, and CAD remains controversial (35–38). Our study underscores that the effects of CCR6 and CXCR4 on CAD might be B cell subtype dependent. Particularly in α-gal sensitized subjects, these chemokine receptors may aggravate CAD through promoting SWM B cells to produce IgE to α-gal. However, further studies with larger cohorts will be needed to validate the link between alpha-gal sensitization and CAD and also to further explore the role of CCR6 and CXCR4 in contributing to class switch and CAD. More detailed studies will also be required to confirm whether implicated pathways are specific for alpha-gal IgE class switch, or could be generalizable to IgE switching to other antigens.

In summary, here we identified that human CCR6+ class-switched memory B cells, but not other B cell subtypes, were associated with α-gal IgE sensitization. Further transcriptome analysis and *in vitro* characterization also revealed that CCR6 and CXCR4, acting via non-chemotactic mechanisms, contributed to IgE isotype switching and cell proliferation of SWM B cells, respectively. In addition, we demonstrated that relatively higher expression of CCR6 and CXCR4 in SWM B cells were associated with CAD severity, independent of traditional CAD risk factors. These findings suggest that therapies that modulate SWM B cells, potentially by targeting CCR6 or CXCR4, hold promise for reducing α-gal IgE production and cardiovascular disease.

## Data Availability Statement

The datasets presented in this study can be found in online repositories. The names of the repository/repositories and accession number(s) can be found below: GSE190570.

## Ethics Statement

The studies involving human participants were reviewed and approved by University of Virginia Institutional Review Board. The patients/participants provided their written informed consent to participate in this study.

## Author Contributions

TP and CM conceived the study. TP designed, performed and analyzed experiments. CM, JW, and LE supervised experiments and provided insights on study designs. YG and RG designed the bioinformatics pipeline for CITEseq analysis. CD performed CITEseq experiments. JV and RS helped with CITEseq data thresholding. TP-M provided ImmunoCAP assays. AT provided patient cohorts. KL supervised CITEseq experiments. HQ performed *in vitro* B cell stimulation experiments. CM supervised the entire study. TP, JW, LE, and CM prepared the manuscript. All authors contributed to and approved the final manuscript.

## Funding

This work was supported by National Institute of Health NIH R01HL 136098 (CM) R01-HL148109 (CM), P01 HL136275 (CM and KL), R21-AI-152447 (LE), R37-AI-20565 (TP-M), and AAAAI Faculty Development Award (JW). This study received assay support from Thermo-Fisher/Phadia. The funder was not involved in the study design, collection, analysis, interpretation of data, the writing of this article, or the decision to submit it for publication.

## Conflict of Interest

The authors declare that the research was conducted in the absence of any commercial or financial relationships that could be construed as a potential conflict of interest.

## Publisher's Note

All claims expressed in this article are solely those of the authors and do not necessarily represent those of their affiliated organizations, or those of the publisher, the editors and the reviewers. Any product that may be evaluated in this article, or claim that may be made by its manufacturer, is not guaranteed or endorsed by the publisher.
