# Supplementary figures and images for: Chemokine Receptor Activation Enhances Memory B Cell Class Switching Linked to IgE Sensitization to Alpha Gal and Cardiovascular Disease
Source: Front Cardiovasc Med. 2022 Jan 13;8:791028. doi: 10.3389/fcvm.2021.791028 (PMC8793803; doi:10.3389/fcvm.2021.791028)

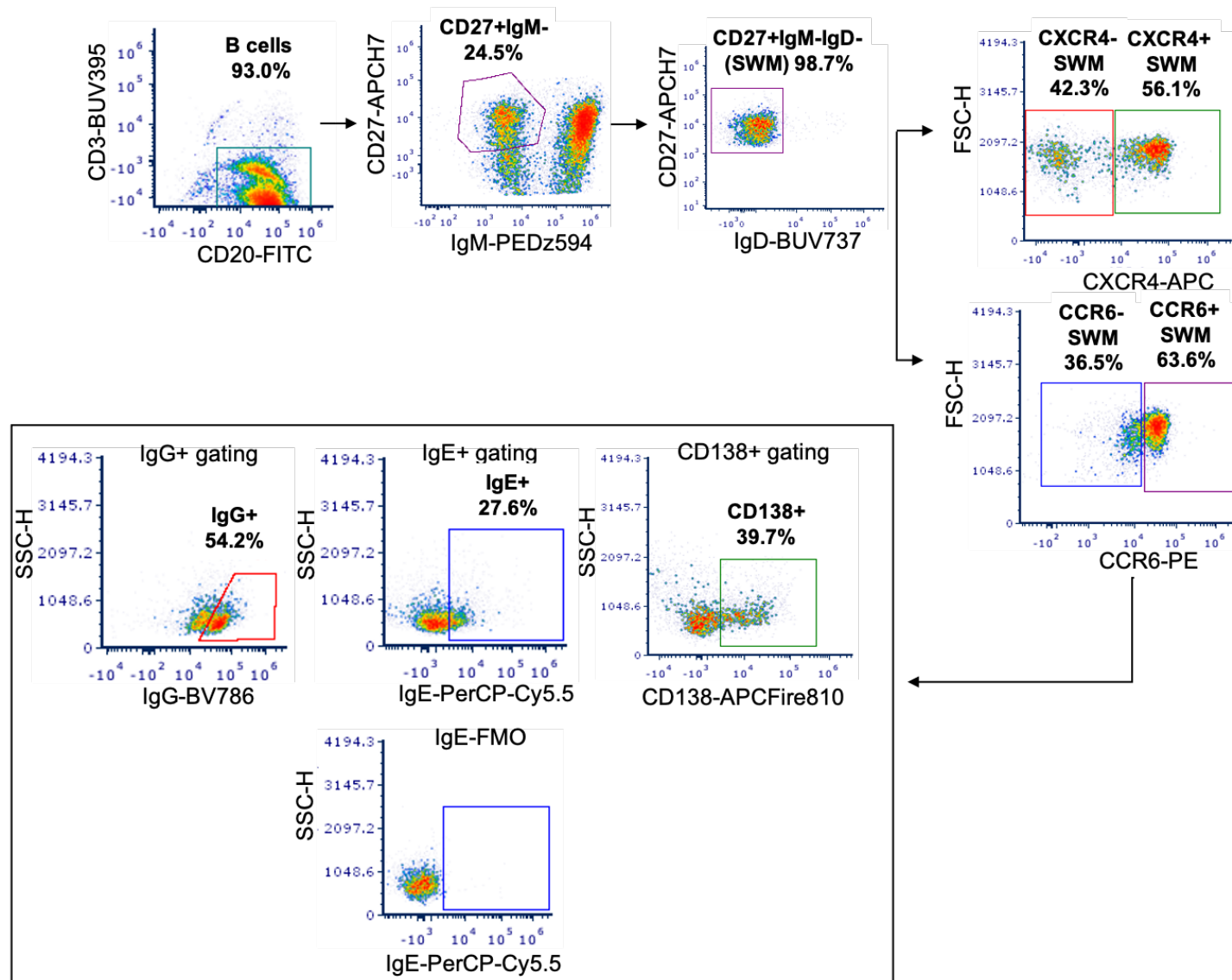

**Supplementary Figure 1: Gating strategy of CXCR4 +/- and CCR6 +/- SWM as well as IgG, IgE and CD238+ populations**

Supplement: Supplementary file 1 [file Data_Sheet_1.PDF]
