# Supplementary material for: Chemokine Receptor Activation Enhances Memory B Cell Class Switching Linked to IgE Sensitization to Alpha Gal and Cardiovascular Disease
Source: Front Cardiovasc Med. 2022 Jan 13;8:791028. doi: 10.3389/fcvm.2021.791028 (PMC8793803; doi:10.3389/fcvm.2021.791028)
Supplement: Supplementary file 3 [file Data_Sheet_3.PDF]

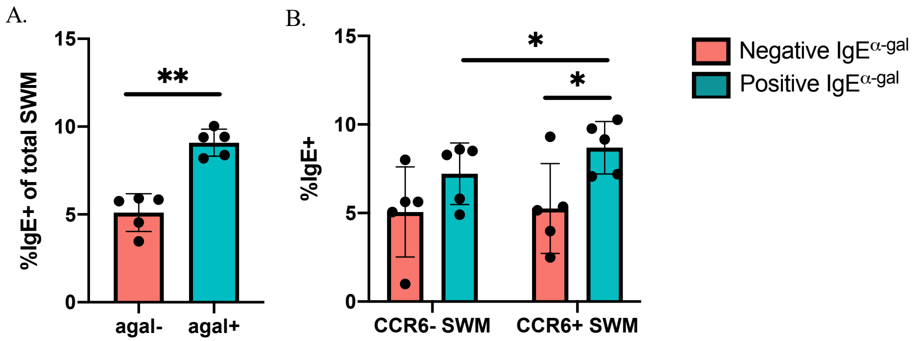

Supplementary Figure 3: Higher level of IgE expressing CCR6+ SWM was observed in subjects with α-gal sensitization. (A-B) Flow cytometry characterization of B cells obtained from CAVA subjects to evaluate percentages of IgE+ of total SWM **(A)** and percentages of IgE+ of CCR6- or CCR6+ SWM **(B)** between subjects with and without α-gal sensitization.
