# Supplementary material for: Chemokine Receptor Activation Enhances Memory B Cell Class Switching Linked to IgE Sensitization to Alpha Gal and Cardiovascular Disease
Source: Front Cardiovasc Med. 2022 Jan 13;8:791028. doi: 10.3389/fcvm.2021.791028 (PMC8793803; doi:10.3389/fcvm.2021.791028)
Supplement: Supplementary file 4 [file Data_Sheet_4.PDF]

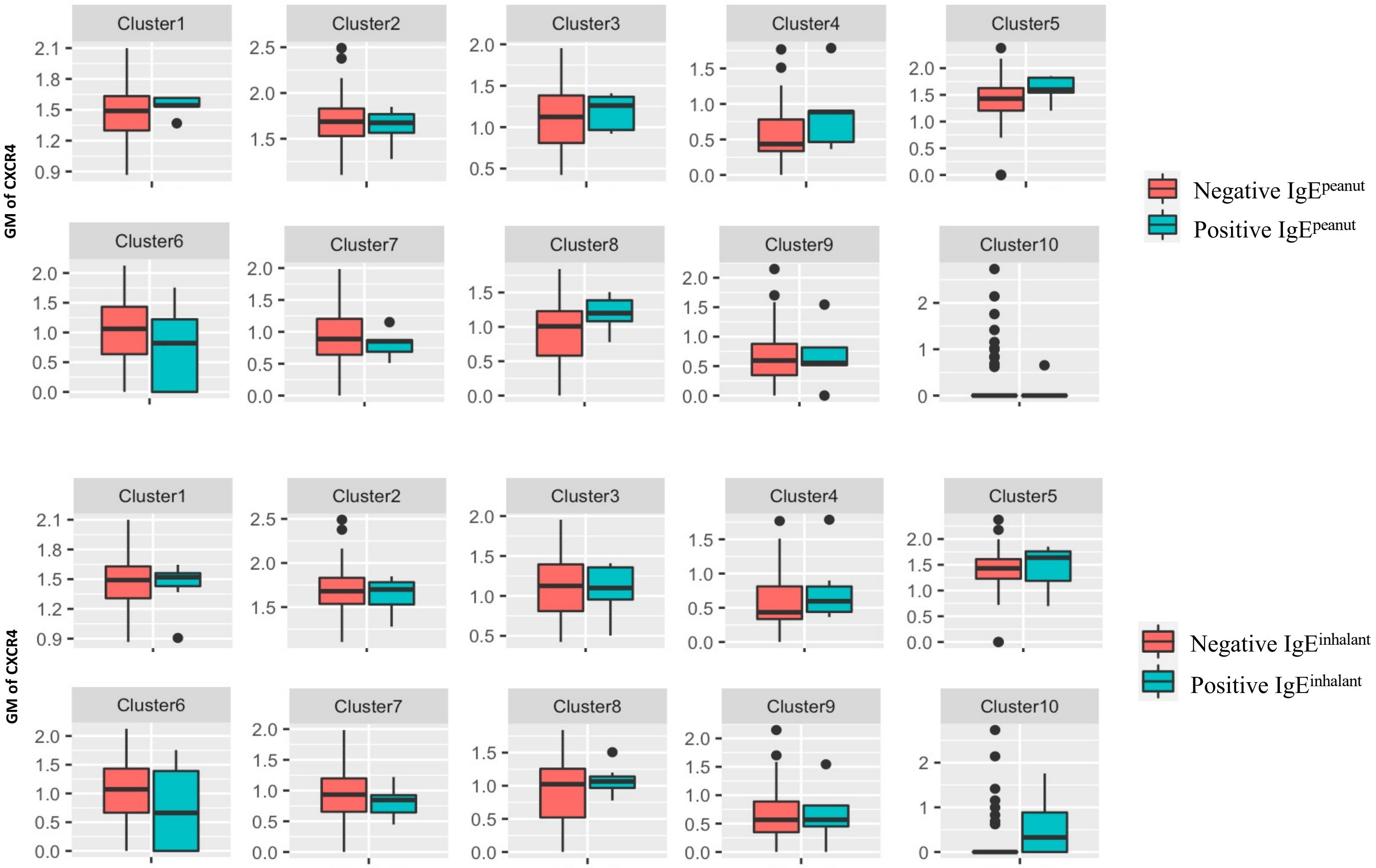

Supplementary Figure 4: CXCR4 expression of 10 B cell subtypes comparing between subjects tested positive and negative for IgE-peanut and IgE-inhalant.
