# Supplementary material for: Chemokine Receptor Activation Enhances Memory B Cell Class Switching Linked to IgE Sensitization to Alpha Gal and Cardiovascular Disease
Source: Front Cardiovasc Med. 2022 Jan 13;8:791028. doi: 10.3389/fcvm.2021.791028 (PMC8793803; doi:10.3389/fcvm.2021.791028)
Supplement: Supplementary file 7 [file Data_Sheet_7.PDF]

Supplementary Table 1: Clinical characteristics of the CAVA cohort subjects with and without α-gal IgE sensitization utilized to perform CITESeq

	IgE to α-gal		
	**Positive (n=17)**	**Negative (n=43)**	**p-value**
Age	62 ± 8.71	64.4 ± 8.83	0.30
BMI	33.68 ± 5.32	31.5 ± 6.62	0.19
Hypertension	58.8%	77.5%	0.17
Diabetes	35.2%	50%	0.36
%Caucasian	100%	92.5%	0.26
% Male	30.7%	11.7%	0.13
Total Cholesterol (mg/dL)	137 ± 33.81	146 ± 38.74	0.42
HDL (mg/dL)	39.4 ± 13.50	41.7 ± 10.21	0.49
LDL (mg/dL)	77 ± 29.08	84 ± 33.21	0.39
IgE to α-gal	1.20 ± 3.99	ND	

ND = not detectable
