# Supplementary material for: Chemokine Receptor Activation Enhances Memory B Cell Class Switching Linked to IgE Sensitization to Alpha Gal and Cardiovascular Disease
Source: Front Cardiovasc Med. 2022 Jan 13;8:791028. doi: 10.3389/fcvm.2021.791028 (PMC8793803; doi:10.3389/fcvm.2021.791028)
Supplement: Supplementary file 8 [file Data_Sheet_8.PDF]

Supplementary Table 2: List of 50 titrated Ab-Oligos antibodies.

Antigen	Clone	Antigen	Clone
CD11b	M1/70	CD24	ML5
CD11c	B-LY6	CD25	2A3
CD123 (IL-3RA)	7G3	CD27	M-T271
CD126 (IL-6R)	M5	CD273	MIH18
CD127 (IL-7R)	HIL-7R-M21	CD274	MIH1
CD137	4B4-1	CD279	MIH4
CD14	MPHIP9	CD3	SK7
CD141	1A4	CD33	WM53
CD142	HTF-1	CD36	CB38 (NL07)
CD152 (CTLA-4)	BNI3	CD38	HIT2
CD154	TRAP1	CD4	SK3
CD16	3G8	CD43	1G10
CD163	GHI/61	CD45RA	HI100
CD183 (CXCR3)	1C6/CXCR3	CD45RO	UCHL1
CD184 (CXCR4)	12G5	CD56	NCAM16.2
CD185 (CXCR5)	RF8B2	CD69	FN50
CD19	SJ25C1	CD8	RPA-T8
CD192 (CCR2)	1D9	CD86	2331(FUN-1)
CD194 (CCR4)	1G1	CD9	M-L13
CD195 (CCR5)	2D7/CCR5	CD95	DX2
CD196 (CCR6)	11A9	HLA-DR (CD74)	G46-6
CD197 (CCR7)	3D12	IgD	IA6-2
CD2	RPA-2.10	IgM	G20-127
CD20	2H7	TLR4	TF901
CD206	19.2	SLAN	hSF6
CD223 (LAG-3)	T47-530		

Supplementary Table 3: List of 26 titrated flow antibodies.

Antigen	Fluorophore	Clone
CD3	BUV395	UCHT1
CD21	BUV496	B-ly4
CD9	BUV563	M-L13
CD45RB	BUV661	16A
IgD	BUV737	IA6-2
CD27	BV421	M-T27I
FcRεI	BV510	AER-37
CD20	Pacific Orange	HI47
IgM	BV570	MHM-88
AID	BV650	EK2-5G9
CD24	BV711	M1/69
CD185	BV750	RF8B2
CD279	BV785	29F.1A12
CD23	BB515	B3B4
IgG	FITC	M1310G05
IgE	PerCP-Cy5.5	MHE-18
TLR4	BB700	TF901
CD196	PE	G034E3
CD80	PE/Dazzle594	2D10
CD62L	PE-Cy5	DREG-56
TLR2	PE-Cy7	TL2.1
CD184	APC	12G5
CD72	Alexa Fluor 647	982405
Live Dead	ZombieNIR	
CD40	APC-R700	5C3
CD138	APC-Fire810	281-2
